# Micropatterned Ultrathin MOF Membranes with Enhanced Molecular Sieving Property

**DOI:** 10.1002/anie.201809872

**Published:** 2018-09-19

**Authors:** Kang Huang, Bo Wang, Song Guo, Kang Li

**Affiliations:** ^1^ Barrer Centre Department of Chemical Engineering Imperial College London London SW7 2AZ UK; ^2^ Department of Biomedical Engineering National University of Singapore 7 Engineering Drive 1 Singapore 117574 Singapore

**Keywords:** membranes, metal–organic frameworks, micropatterned ceramics, molecular sieving, UiO-66

## Abstract

Metal–organic frameworks (MOFs) are attractive crystalline materials for membranes due to their diverse crystalline pore structures and molecular separation properties. However, the fabrication cost is relatively high compared to conventional polymeric membranes. The concern of the cost could be eased if they are part of a value‐added device, for example, as the key separation unit in a lab‐on‐a‐chip device. This study demonstrates the feasibility of miniaturization of MOF membranes by patterning the membrane surface, a necessary step for MOF membranes to be used in compact devices. Water‐stable ultrathin UiO‐66 membranes with a thickness down to 250 nm on a substrate with a complex pattern were grown. The patterned membranes showed a 100 % improvement in the apparent permeation flux over conventional flat‐UiO‐66 membranes without compromising the molecular separation property, indicating the complexity of a surface would not be a formidable obstacle to the MOF membrane fabrication.

Metal–organic frameworks (MOFs),^1^ assembled by metal centers and organic linkers, are an attractive class of crystalline microporous materials that have shown great potential in various fields such as gas storage,^2^ catalysis,^3^ sensing,^4^ and adsorptive separation.^5^ Considering their diversities of crystalline pore structures and versatile chemical and physical properties, one of the most efficient and important approaches is to utilize them in the form of thin films, which enable them to realize maximum usage as molecular separation units with minimum usage of MOF materials, which have been intensively demonstrated previously.^6^ In real applications, a high throughput of MOF membranes (such as permeance or selectivity) is essential to make them economically viable. Vast efforts have been made to continuously develop new chemistry and structures of MOF membranes to reach such a target,^7^ but there is still a very large gap between the state of the art and the expected profitable membrane performance, and currently large‐scale industrial application of MOF membranes is economically unfeasible.

Incorporating MOF membranes into devices or instruments could be a promising approach to leverage their potential in real applications, if the MOF membranes can be miniaturized. Miniaturized separation units can play a key role in a lab‐on‐a‐chip device for fast prototyping,^8^ for example, in a micro‐membrane reactor for the design of new chemical processes or the separation of expensive pharmaceutical products,8a,8b or being used as a selective barrier in a sensor.8c MOF membranes can provide sharper molecule‐level separation and much higher throughput compared to polymeric counterparts,6a–6g, 7 making them an ideal selection for such purposes. In miniature separation units, the membrane area to volume ratio needs to be maximized to reach a high throughput in a given volume, which is crucial to the overall efficiency of a device. Hollow fiber geometry may be a good choice as it provides very high membrane area per volume,^9^ but in practice, it would be difficult to assemble them into micro‐devices because of its special construction and the weak mechanical property. Alternatively, patterning the membrane surface could be an attractive approach to increase the effective membrane area whilst easing membrane assembly and remaining strong mechanically, which has been studied by computational fluid dynamics simulations and verified experimentally in polymeric membranes.^10^ It is however difficult for polycrystalline membranes such as zeolite and MOF membranes, and no success on patterned zeolite or MOF membranes has been previously reported. Although there were a few studies employing patterned MOFs,^11^ they were loosely packed particulates11b–11d or deposited on impermeable substrates11e–11j for catalysis, sensor and adsorptive separation, and were not useful for membrane‐based molecular separation.

Herein, for the first time, we successfully demonstrate the growth of continuous zirconium‐MOF UiO‐66 (UiO stands for University of Oslo) membranes on patterned porous yttria‐stabilized zirconia (YSZ) ceramic substrates by a bottom‐up procedure. UiO‐66 is a MOF material with excellent chemical and thermal stabilities, and it is highly stable in water compared to other MOFs, such as ZIF‐8, MOF‐5, HKUST‐1.^12^ The recent development of continuous UiO‐66 membranes showed a great potential in value‐added solvent dehydration process.9d The patterned UiO‐66 membranes in this study exhibited molecular separation property and enhanced performance, which demonstrates the feasibility of MOF membranes developed as a micro‐platform for molecular separation.

In this work, we employed lithographic moulding,^13^ followed by a phase‐inversion technique,9b,9c to prepare patterned porous YSZ substrates. Figure 1 a illustrates how the patterned ceramic substrates were fabricated. First, soft polydimethylsiloxane (PDMS) with designed patterns was prepared by the lithography technique and used as a mould. A suspension composed of the solvent, YSZ ceramic powder, the polymer binder, as well as the dispersant, was poured onto the PDMS mould and replicated the patterns on the mould surface. Then a vacuum was used to remove bubbles and facilitate penetration of the suspension, and a casting knife was used to obtain an even, smooth top surface. The moulded ceramic suspension was then immersed in water to finish the phase‐inversion process. The resultant patterned green ceramic substrates (unsintered ceramic) were peeled off from the PDMS mould and then dried at room temperature. Eventually, the green substrates were sintered at 1170 °C in the air for 5 h.

**Figure 1:**
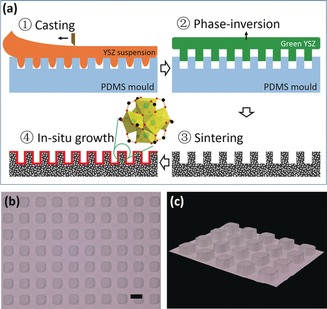
a) Diagram of patterned YSZ ceramic substrates and UiO‐66 membranes prepared. b) Optical microscope image of the array of cuboids on a patterned YSZ substrate (scale bar: 100 μm) and c) the corresponding 3D display image.

Figure 1 b,c shows the array of cuboids on a patterned YSZ substrate. The length, width, and height of the cuboids are about 75, 75, and 32 μm, respectively, and the space between neighbour cuboids is about 60 μm. The thickness of sintered ceramic substrate is about 0.7 mm. Compared to the PDMS mould, the ceramic substrate showed shrinkage of 70±5 %, which was caused by the phase inversion and sintering. Scanning electron microscope (SEM) image (Figure S1 a in the Supporting Information) showed a porous surface on the substrate, and the porosimetry analysis determined that the average pores size was about 90 nm. Furthermore, finger‐like voids formed due to the Rayleigh–Taylor instability during the phase inversion can be observed in the cross‐sectional view (Figure S1 b in the Supporting Information), which are favorable for reducing the mass transport resistance.

The UiO‐66 membrane on the substrate was synthesized by an in situ hydrothermal method (Figure S2 in the Supporting Information). After careful adjustment of the preparation process, the patterned surface was entirely covered by a well‐intergrown UiO‐66 thin film free of visible defects (Figure 2 a), including the corners of the cuboids (see close‐up SEM images in Figure 2 b,c and S3 in the Supporting Information). The thickness of the thin film ranged from about 300 to about 900 nm depending on the location of the surface (Figure 2 d,e), where the top parts were thicker than the valley parts, but were all thinner than most of the reported MOF membranes.6a–6g, 7 The X‐ray diffraction (XRD) pattern (Figure 2 f) and the attenuated total reflectance Fourier transform infrared spectra (ATR‐FTIR, Figure S4 in the Supporting Information) confirmed that the prepared membrane was non‐oriented UiO‐66 polycrystalline phase free of impurities.

**Figure 2:**
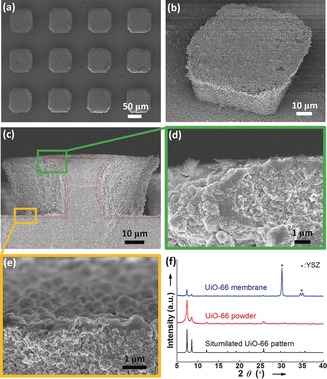
a) Top view SEM image of cuboidal UiO‐66 membranes. b) Close‐up SEM image of a single cuboid. c)–e) Cross‐sectional SEM images of cuboidal UiO‐66 membranes. The red dotted line highlights the continuous polycrystalline layer conforms to the outline of the patterned substrates. f) XRD pattern of the prepared membrane.

The features of the patterns on the substrate can be easily controlled by the PDMS mould. Figure 3 exhibits the YSZ substrates of the other two patterns, namely channels with a rectangular cross section (Figure 3 a) and short cylinders (Figure 3 b). SEM images (Figure 3 c and 3d) indicate that these patterns are fully covered by crystals without visible defects after the same synthesizing process. XRD patterns (Figure S5 in the Supporting Information) and ATR‐FTIR spectra (Figure S4 in the Supporting Information) also confirm that they are the UiO‐66 polycrystalline phase. Same as the cuboid‐patterned samples, the trend of the thickness change was also observed in these two types of membranes. For the membrane with cylinders, the thickness of the UiO‐66 thin film was the thinnest at the valleys, less than 250 nm; while the thickness increased to 1.0 μm at the top of the cylinders (Figure S6 in the Supporting Information). Such a trend can be attributed to the concentration profile of reactants over the patterned surface during the in situ hydrothermal synthesis process. As the formation of the thin film consumes the reactants, a concentration gradient will be built up near the surface, and the continuous supply of reactants relies on the diffusion alone. The diffusion towards the valley surface would be slower than the top surface, as it faced the competition from sidewalls, which led to slower delivery of the reactants to the valley surface and thus slower growth of the thin film. The competing effect would be more visible when the distance between sidewalls is reduced. This is confirmed by a comparative study with two channeled surfaces: the thickness of the thin film at valleys reduced from ca.700 to ca.450 nm when the width of the channels reduced from ca.150 to ca.80 μm (see Figure S7 in the Supporting Information). Similarly, when the depth of the channels reduced from ca.50 to ca.10 μm, the thickness increased from ca.460 to ca.830 nm owing to less competing effects from the sidewalls (Figure S8 in the Supporting Information).

**Figure 3:**
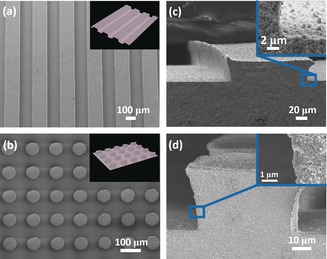
SEM images of UiO‐66 membranes with channeled and cylindrical patterns: a),b) Top view; c),d) cross‐sectional view. The insets in (a) and (b) are corresponding 3D display images. The insets in (c) and (d) are enlarged cross‐sectional views.

As a prototypical Zr‐MOF with a formula of Zr_6_O_4_(OH)_4_(BDC)_6_ (BDC=1,4‐benzene‐dicarboxylate) and fcu topology,^12^ UiO‐66 membranes have been proven to be excellent candidates for organic solvent dehydration owing to the hydrophilic nature offered by the hydroxy groups.9d Herein, the quality and separation performance of the patterned UiO‐66 membranes were examined through butanol dehydration by the pervaporation process. Figure 4 summaries the separation performance of the three patterned UiO‐66 membranes. As a comparison, a flat membrane without pattern was synthesized using the same method and its separation performance is also shown. All the membranes exhibited excellent dehydration performance. The water concentrations were improved from 10 wt % to above 99 wt % in the permeate, corresponding to a separation factor of over 1000, which confirmed that the membranes were of defect‐free quality and sufficient for molecular level separation. The results are encouraging, as the complex patterns, and especially the corners were expected to impact negatively on the quality of the membranes, but here the patterned membranes showed equal quality with the unpatterned membrane. Furthermore, the flux of the patterned UiO‐66 membranes was considerably higher than the unpatterned membrane. Notably, the cuboidal UiO‐66 membrane showed a flux increase of more than 100 %, reaching 2.96 kg m^−2^ h with a separation factor of 1102 at room temperature, which is superior to most of the reported membranes (Table S1 in the Supporting Information). The enhancement of the flux can be partially attributed to the increased membrane area of the patterned surface. Compared to the unpatterned membrane, the geometrical membrane area of the cuboidal, cylindrical, and channeled membranes increased about 65, 50, and 30 %, respectively. Another factor contributing to the increased flux could be the reduced average thickness of the patterned membranes, with the thinnest parts only about 300 nm, remarkably thinner than the unpatterned membrane, which was about 1.0 μm. The separation performance of the channeled membranes with different depths showed that even with the same type of patterns, the flux also changed corresponding to the change in surface area and membrane thickness, demonstrating the importance of these two factors (Figure S9 in the Supporting Information). It should be noted that during the pervaporation process in the current study, the flow pattern of the feed solution was not optimized to reduce concentration polarization‐related flux reduction, and thus the advantages of the patterned surface were not fully elaborated. It is very likely that the optimization of the UiO‐66 membrane module would gain even better separation performance, as one would expect from previous studies.^10^ To further increase the throughput, patterns with larger height‐to‐width ratios can be adopted to achieve higher geometric membrane area.

**Figure 4:**
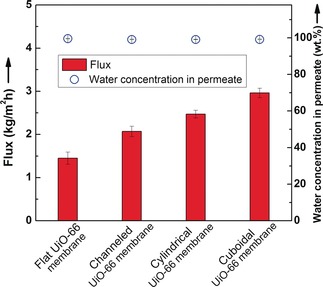
Separation performance of UiO‐66 membranes with different patterns.

In summary, we have demonstrated, for the first time, the successful growth of defect‐free MOF membranes on patterned substrates with high complexity. The concave part of the patterned surface experienced slower MOF growth, and thinner membranes were resulted compared to the outmost surface. This has led to a reduced average membrane thickness, which contributed to higher permeation flux together with the increased membrane area of the patterned membranes. Besides the improved separation performance of the patterned membranes, this study also indicates the feasibility of growing high‐quality MOF thin films on complex surfaces despite the existence of highly curved corners. This allows us to design and realize miniature molecular separation units for portable instruments and lab‐on‐a‐chip devices, thanks to the high area to volume ratio achievable with patterned surfaces.

## Conflict of interest

The authors declare no conflict of interest.

## Supporting information

As a service to our authors and readers, this journal provides supporting information supplied by the authors. Such materials are peer reviewed and may be re‐organized for online delivery, but are not copy‐edited or typeset. Technical support issues arising from supporting information (other than missing files) should be addressed to the authors.

Supplementary
