# Supplementary material for: Micropatterned Ultrathin MOF Membranes with Enhanced Molecular Sieving Property
Source: Angew Chem Int Ed Engl. 2018 Sep 19;57(42):13892–6. doi: 10.1002/anie.201809872 (PMC6334230; doi:10.1002/anie.201809872)
Supplement: Supplementary file 1 — Supplementary [file ANIE-57-13892-s001.pdf]

Angewandte Chemie
Eine Zeitschrift der Gesellschaft Deutscher Chemiker

Supporting Information

Micropatterned Ultrathin MOF Membranes with Enhanced Molecular Sieving Property

*Kang Huang, Bo Wang, Song Guo, and Kang Li**

anie_201809872_sm_miscellaneous_information.pdf

Experimental

Preparation of UiO-66 membranes on the patterned YSZ ceramic substrates

A controlled in-situ hydrothermal method [1-2] was used to grow UiO-66 membranes on the patterned YSZ ceramic substrates. As shown in Figure S2, the patterned YSZ ceramic substrate was placed, with the patterned surface downwards, in a Teflon-lined stainless steel autoclave (100 ml) filled with the synthetic solution. The typical synthetic solution was prepared by dissolving $ZrCl_4$ (0.419 g, Sigma-Aldrich) and BDC ligands (0.299 g, Sigma-Aldrich) in N,N-dimethylformamide (70 mL, VWR), followed by mixing with 0.032 g deionized water. Then, the autoclave was kept at 120 °C for 48 h. After cooling, the membrane was washed with DMF and dried under ambient condition. The UiO-66 powder was also collected from the reacted solution and washed with ethanol for later characterizations.

Characterization

Morphological characterizations were carried out on LEO Gemini 1525 scanning electronic microscope (SEM, Tokyo, Japan). The samples were coated with 10-nm thick chromium before observations. Crystallography analysis was done with a Panalytical Xpert X-ray diffraction apparatus using Cu Kα radiation (λ = 0.154 nm) at 40 kV and 20 mA. FTIR-ATR spectra were recorded using an FTIR spectrophotometer (Spectrum 100, PerkinElmer) over the wavelength range of 4000-600 cm^{-1}. The average pore size of the patterned YSZ ceramic substrate was determined by the gas-liquid displacement method using a capillary flow porometer (POROLUX 1000, POROMETER nv, Belgium)). Optical microscope images were recorded by a digital microscope (VHX-900F, KEYENCE).

Pervaporation experiments

Dehydration of butanol by pervaporation was conducted on a home-made setup at room temperature.[1] The membrane was immersed in the feed tank filled with a butanol-water mixture with 10 wt% water. The pressure of the permeate side was maintained at

200 Pa using a vacuum pump. The permeated vapor was condensed in a liquid nitrogen cold trap. Finally, the concentrations of the sample were determined by gas chromatography.

The PV performance of a membrane is usually expressed in terms of the permeation flux J (g/m^{2}h) and separation factor α. The total flux J was calculated by the weight gain in the cold trap:

$$J = \frac{M}{At} \qquad \text{Eq. (1)}$$

where M is the total mass increase (g) over the collection time t (h), and A is the apparent membrane area (m^2).

The separation factor (a) was defined as follows:

$$\alpha_{i,j} = \frac{y_i / y_j}{x_i / x_j} \qquad \text{Eq. (2)}$$

where x and y are mass fractions of components on the feed and permeate side, respectively.

Figure S1

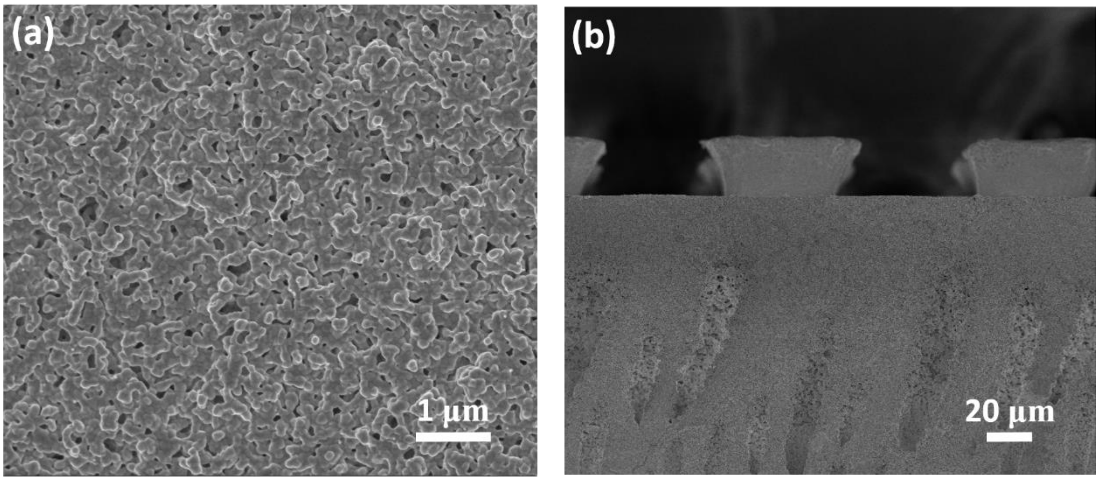

Figure S1. SEM images of the patterned YSZ ceramic substrate: a) surface and b) cross-section.

Figure S2

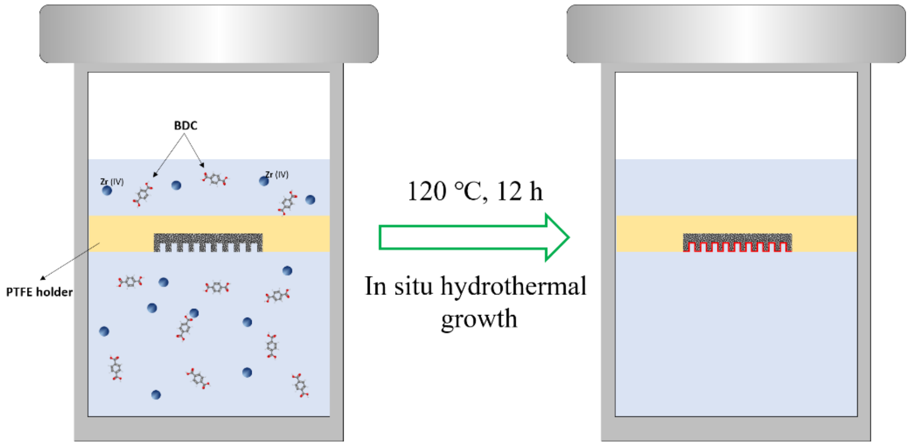

Figure S2. Schematic of the in-situ hydrothermal MOF growing method.

Figure S3

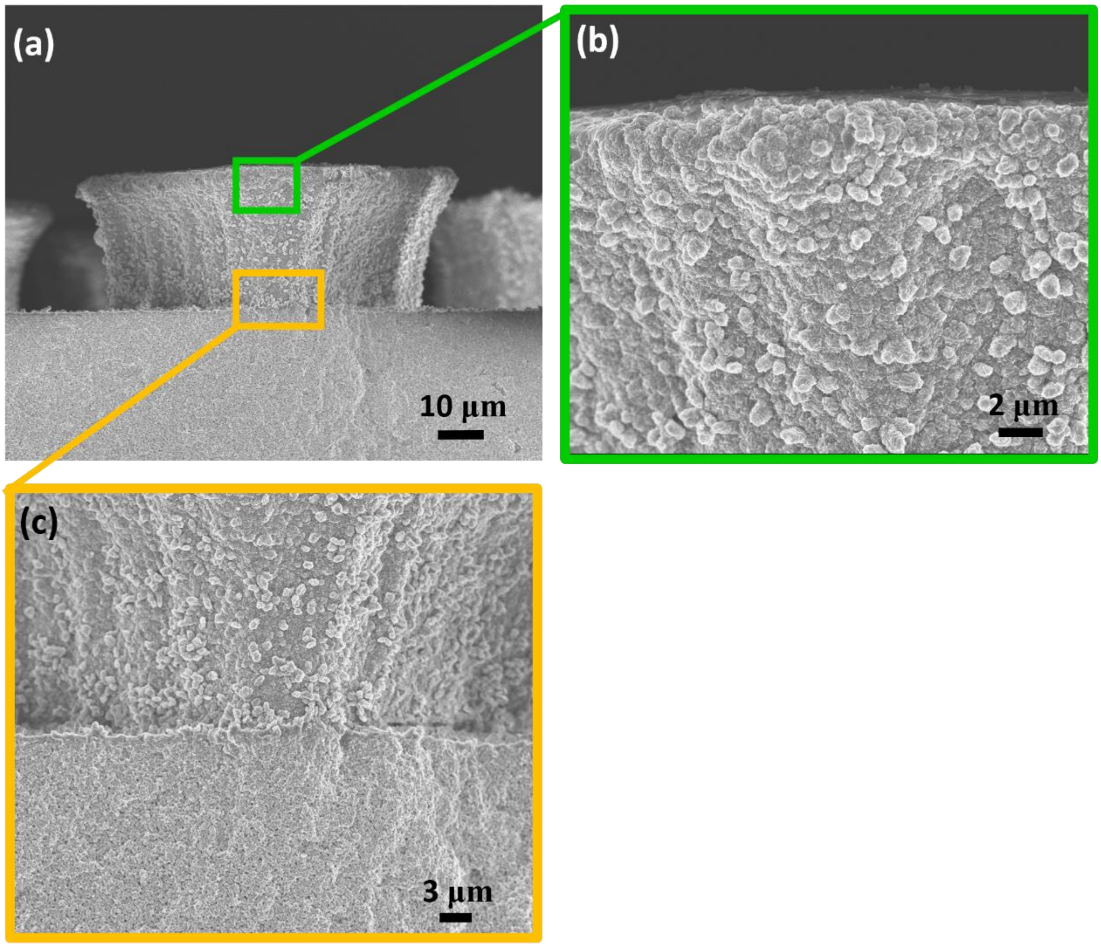

Figure S3. SEM images of the cuboidal UiO-66 membrane, showing a continuous UiO-66 layer covering the substrate surface.

Figure S4

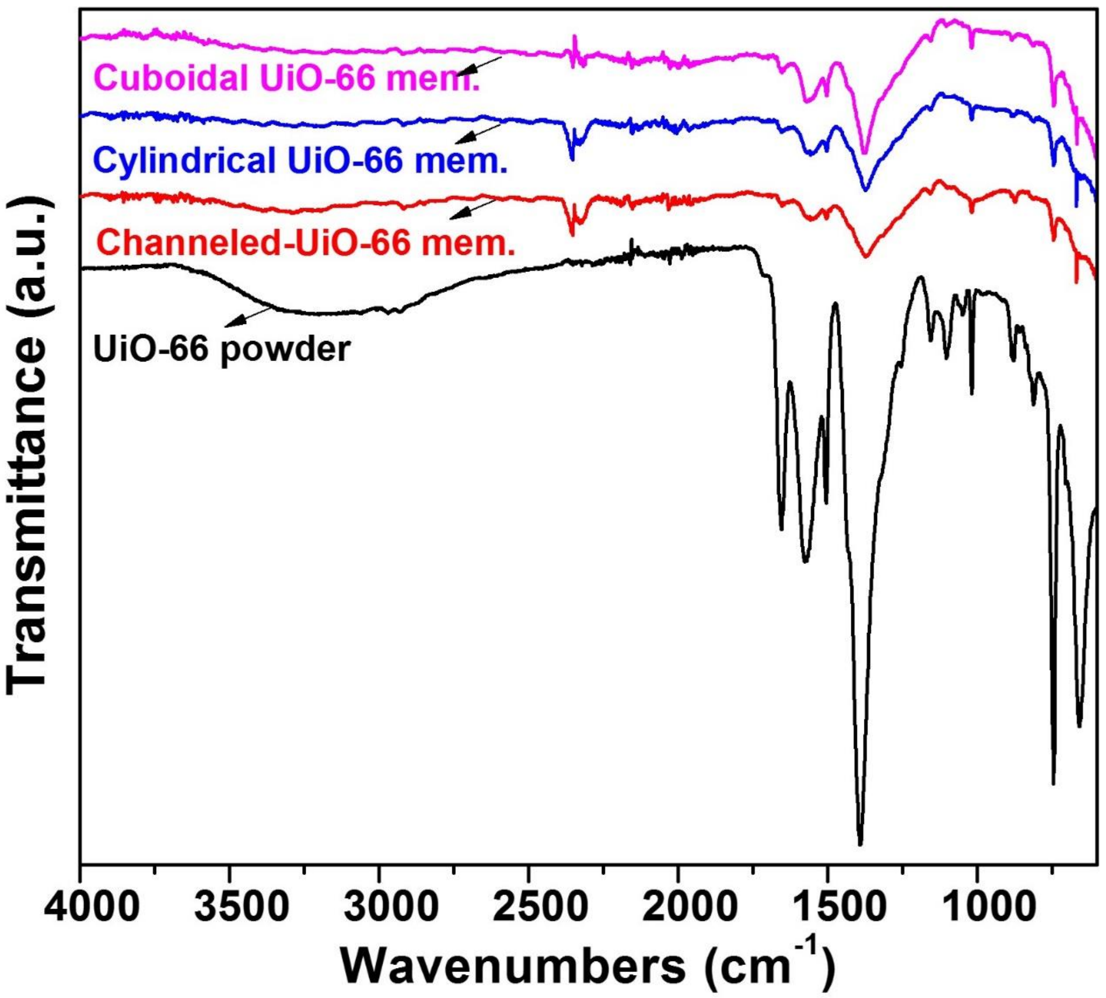

Figure S4. ATR-FTIR results of the prepared UiO-66 membranes.

Figure S5

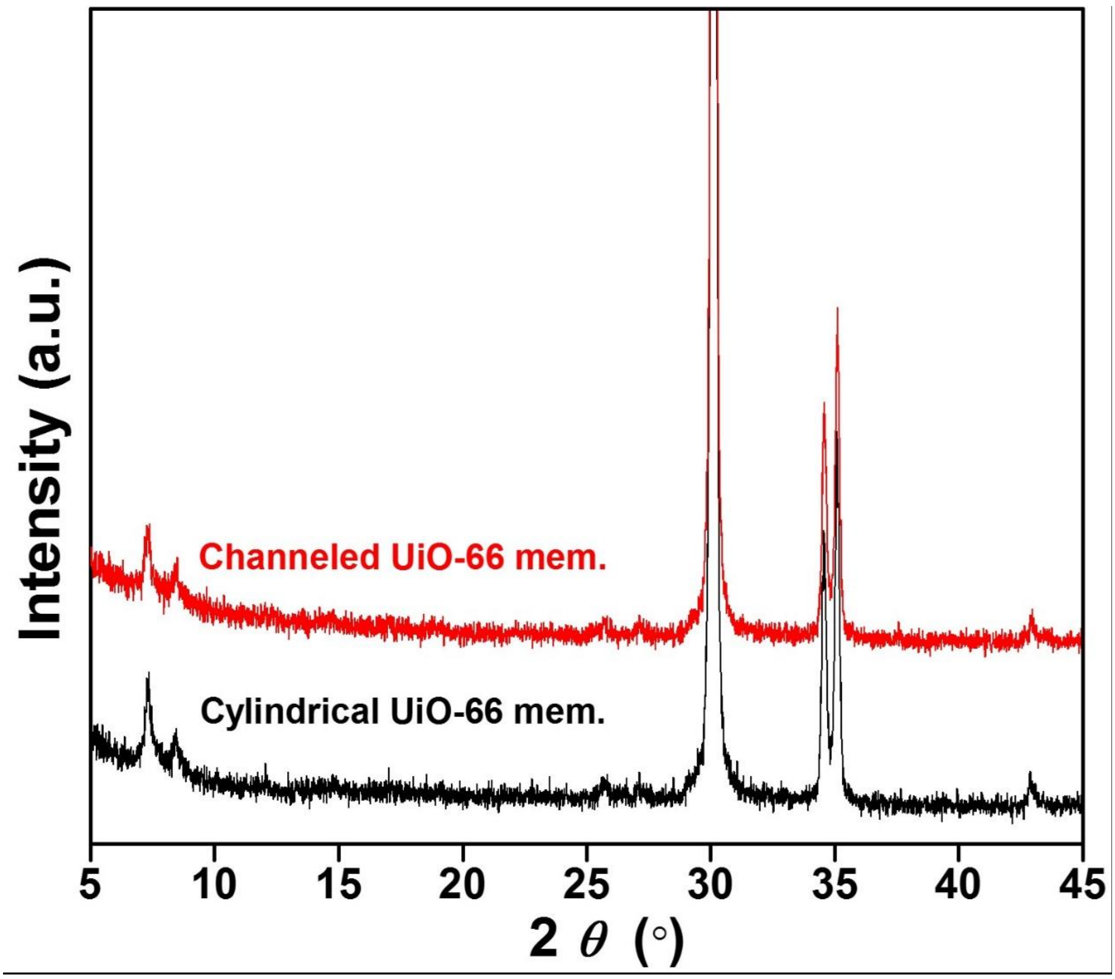

Figure S5. XRD pattern of the prepared membranes with channeled and cylindrical patterns**.**

Figure S6

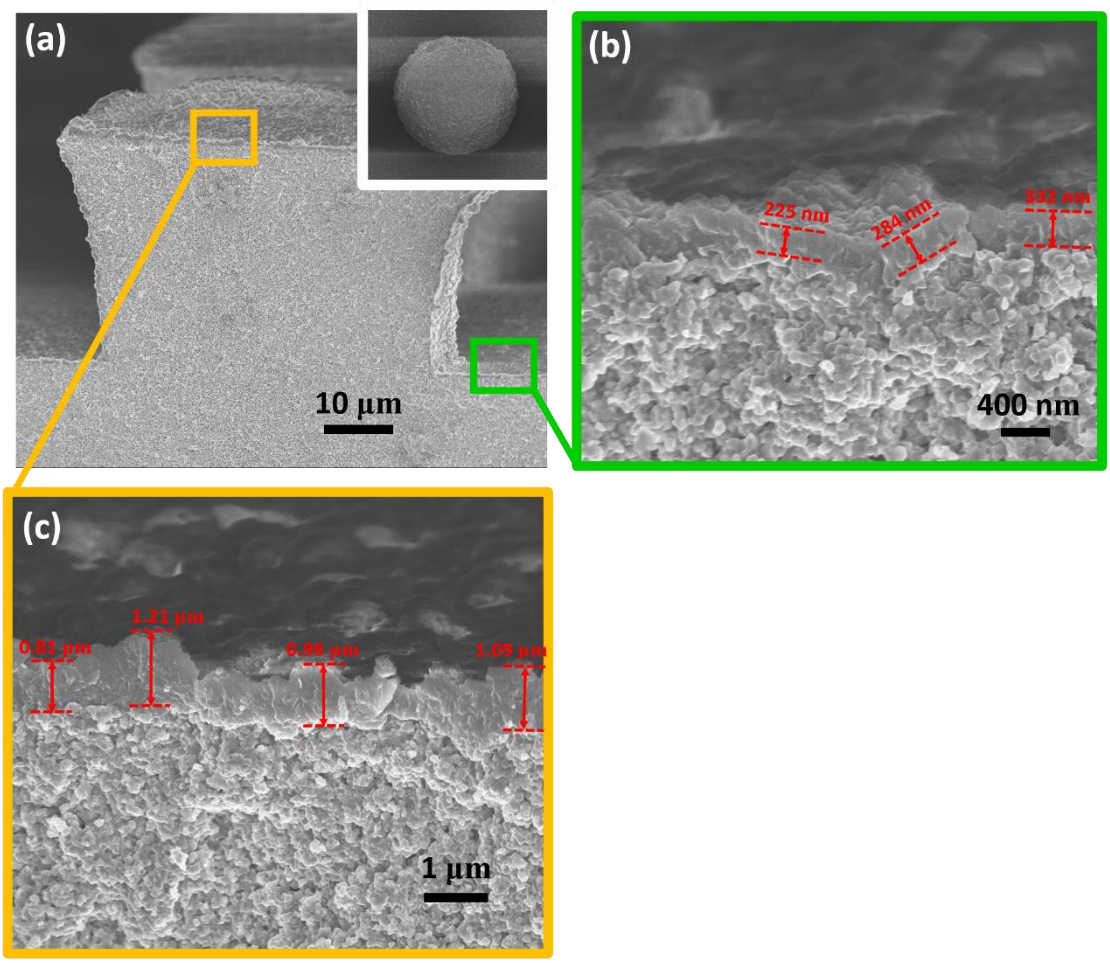

Figure S6. Cross-sectional SEM images of the cylindrical UiO-66 membrane.

Figure S7

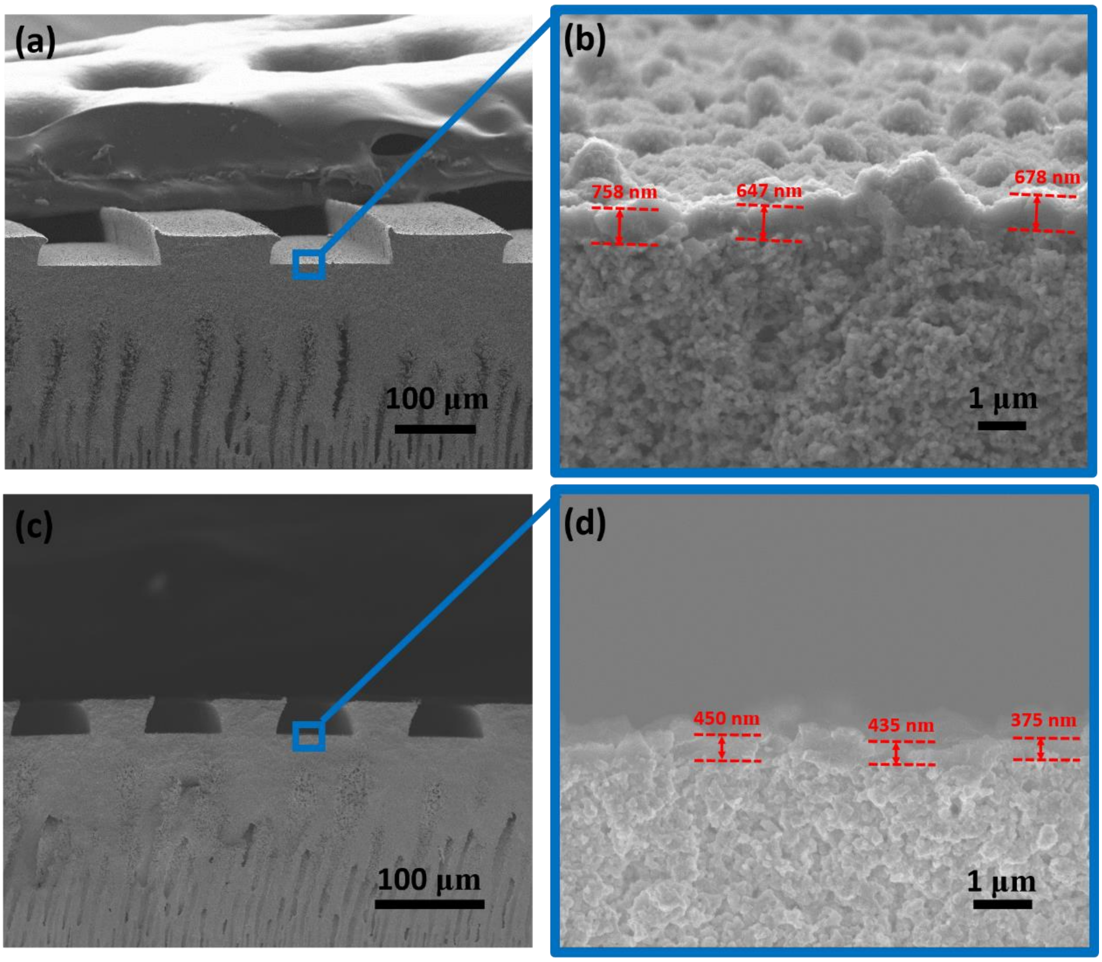

Figure S7. SEM images of two channeled UiO-66 membranes with different width: (a, b) 150 µm; (c, d) 80 µm.

Figure S8

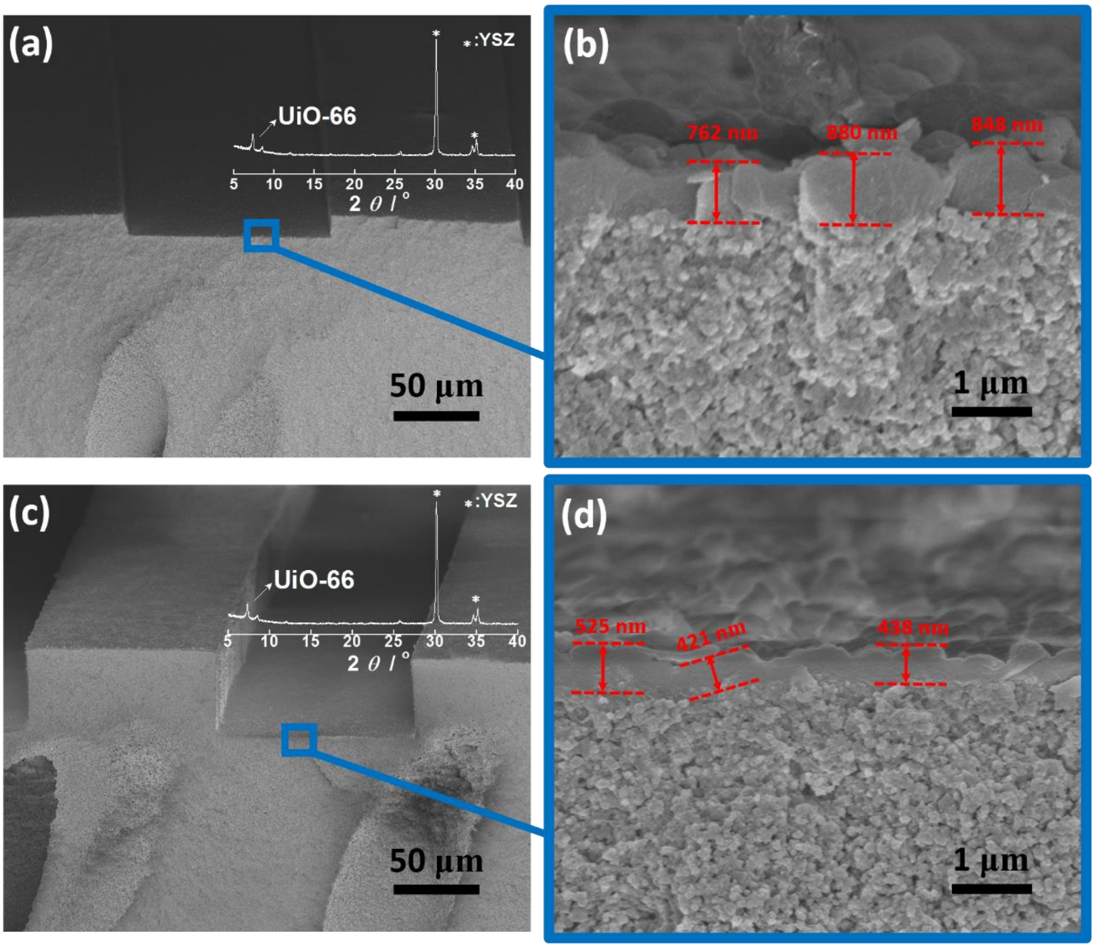

Figure S8. SEM images of two channeled UiO-66 membranes with different depth: (a, b) 10 μm; (c, d) 50 μm.

Figure S9

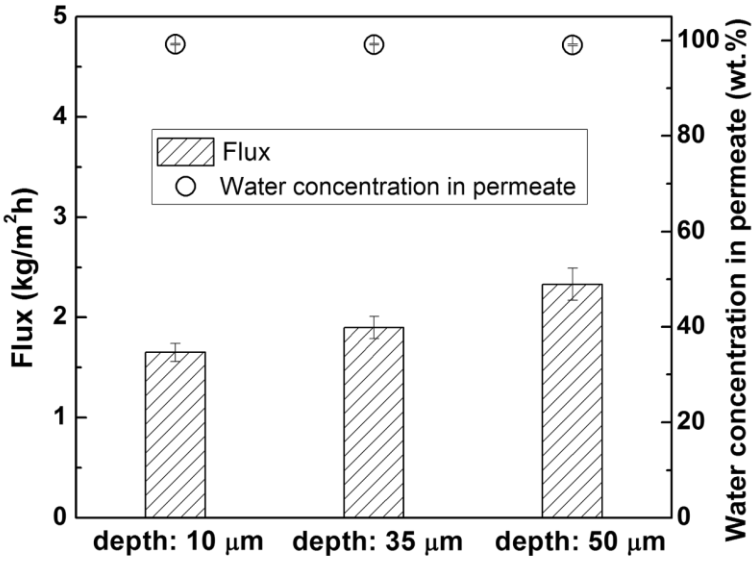

Figure S9. Separation performance of UiO-66 membranes with different depth.

Table S1. Comparison of butanol dehydration separation performance.

Membranes	Feed content (wt %)	Flux (g/m^2h)	Separation factor	Ref.
PVA/ceramic hollow fiber	95 (n-Butanol)	1,000	450	[3]
PVA/PAN	95 (n-Butanol)	250	350	[4]
Pervap®2510	95 (n-Butanol)	700	180	[5]
6FDA-ODA-NDA/Ultem® 1010	85 (n-Butanol)	390	2,518	[6]
sPPSU	85 (n-Butanol)	30	11	[7]
PVA-CS/ceramic	90 (i-Butanol)	1,116	1,000	[8]
P84/ceramic	95 (n-Butanol)	1,400	931	[9]
Matrimid hollow fiber	85 (t-Butanol)	630	491	[10]
PI/PEI dual-layer hollow fiber	85 (n-Butanol)	846	1,174	[11]
PAA/polyethyleneimine	95 (t-Butanol)	769	481	[12]
QP4VP/CMCNa	90 (n-Butanol)	2,241	1,116	[13]
ZIF-8/PBI	85 (n-Butanol)	81	3,417	[14]
TR-PBO	90 (n-Butanol)	58	390	[15]
CS	96 (t-Butanol)	210	2,657	[16]
Methylated silica	94 (n-Butanol)	1,500	1,000	[17]
Tubular silica	95 (n-Butanol)	2,300	680	[18]
Tubular silica	95 (n-Butanol)	3,000	250	[19]
Hydrophobic silica	5 (n-Butanol)	1,500	15	[20]
Zeolite LTA	95 (i-Butanol)	1,210	2,811	[21]
Cuboidal UiO-66	90 (n-Butanol)	2,960	1,102	This work

References

1. Liu X, Wang C, Wang B, Li K: **Novel organic-dehydration membranes prepared from zirconium metal-organic frameworks**. *Adv Funct Mater* 2017, **27**:1604311.
2. Liu X, Demir NK, Wu Z, Li K: **Highly water-stable zirconium metal-organic framework UiO-66 membranes supported on alumina hollow fibers for desalination**. *J Am Chem Soc* 2015, **137**:6999-7002.
3. Peters T, Poeth C, Benes N, Buijs H, Vercauteren F, Keurentjes J: **Ceramic-supported thin PVA pervaporation membranes combining high flux and high selectivity; contradicting the flux-selectivity paradigm**. *J Membr Sci* 2006, **276**:42-50.
4. Schehlmann MS, Wiedemann E, Lichtenthaler RN: **Pervaporation and vapor permeation at the azeotropic point or in the vicinity of the LLE boundary phases of organic/aqueous mixtures**. *J Membr Sci* 1995, **107**:277-282.
5. Guo WF, Chung T-S, Matsuura T: **Pervaporation study on the dehydration of aqueous butanol solutions: a comparison of flux vs. permeance, separation factor vs. selectivity**. *J Membr Sci* 2004, **245**:199-210.
6. Widjojo N, Chung T-S: **Pervaporation dehydration of C2–C4 alcohols by 6FDA-ODA-NDA/Ultem® dual-layer hollow fiber membranes with enhanced separation performance and swelling resistance**. *Chem Eng J* 2009, **155**:736-743.
7. Tang Y, Widjojo N, Shi GM, Chung T-S, Weber M, Maletzko C: **Development of flat-sheet membranes for C1–C4 alcohols dehydration via pervaporation from sulfonated polyphenylsulfone (sPPSU)**. *J Membr Sci* 2012, **415**:686-695.
8. Zhu Y, Xia S, Liu G, Jin W: **Preparation of ceramic-supported poly (vinyl alcohol)–chitosan composite membranes and their applications in pervaporation dehydration of organic/water mixtures**. *J Membr Sci* 2010, **349**:341-348.
9. Kreiter R, Wolfs DP, Engelen CW, van Veen HM, Vente JF: **High-temperature pervaporation performance of ceramic-supported polyimide membranes in the dehydration of alcohols**. *J Membr Sci* 2008, **319**:126-132.
10. Guo WF, Chung T-S: **Study and characterization of the hysteresis behavior of polyimide membranes in the thermal cycle process of pervaporation separation**. *J Membr Sci* 2005, **253**:13-22.
11. Wang Y, Goh SH, Chung TS, Na P: **Polyamide-imide/polyetherimide dual-layer hollow fiber membranes for pervaporation dehydration of C1–C4 alcohols**. *J Membr Sci* 2009, **326**:222-233.
12. Zhang G, Song X, Ji S, Wang N, Liu Z: **Self-assembly of inner skin hollow fiber polyelectrolyte multilayer membranes by a dynamic negative pressure layer-by-layer technique**. *J Membr Sci* 2008, **325**:109-116.
13. Liu T, An Q-F, Zhao Q, Lee K-R, Zhu B-K, Qian J-W, Gao C-J: **Preparation and characterization of polyelectrolyte complex membranes bearing alkyl side chains for the pervaporation dehydration of alcohols**. *J Membr Sci* 2013, **429**:181-189.
14. Shi GM, Yang T, Chung TS: **Polybenzimidazole (PBI)/zeolitic imidazolate frameworks (ZIF-8) mixed matrix membranes for pervaporation dehydration of alcohols**. *J Membr Sci* 2012, **415**:577-586.
15. Ong YK, Wang H, Chung T-S: **A prospective study on the application of thermally rearranged**

acetate-containing polyimide membranes in dehydration of biofuels via pervaporation. *Chem Eng Sci* 2012, **79**:41-53.

16. Biduru S, Sridhar S, Suryanarayana Murthy G, Mayor S: **Pervaporation of tertiary butanol/water mixtures through chitosan membranes cross - linked with toluylene diisocyanate**. *J Chem Technol Biot* 2005, **80**:1416-1424.
17. Bettens B, Verhoef A, van Veen HM, Vandecasteele C, Degrève J, Van der Bruggen B: **Pervaporation of binary water–alcohol and methanol–alcohol mixtures through microporous methylated silica membranes: Maxwell–Stefan modeling**. *Comput Chem Eng* 2010, **34**:1775-1788.
18. Verkerk A, Van Male P, Vorstman M, Keurentjes J: **Properties of high flux ceramic pervaporation membranes for dehydration of alcohol/water mixtures**. *Sep Purif Technol* 2001, **22**:689-695.
19. Cuperus FP, van Gemert RW: **Dehydration using ceramic silica pervaporation membranes—the influence of hydrodynamic conditions**. *Sep Purif Technol* 2002, **27**:225-229.
20. Paradis GG, Shanahan DP, Kreiter R, van Veen HM, Castricum HL, Nijmeijer A, Vente JF: **From hydrophilic to hydrophobic HybSi® membranes: A change of affinity and applicability**. *J Membr Sci* 2013, **428**:157-162.
21. Huang B, Liu Q, Caro J, Huang A: **Iso-butanol dehydration by pervaporation using zeolite LTA membranes prepared on 3-aminopropyltriethoxysilane-modified alumina tubes**. *J Membr Sci* 2014, **455**:200-206.
